# Litter decomposition and nutrient release are faster under secondary forests than under Chinese fir plantations with forest development

**DOI:** 10.1038/s41598-023-44042-5

**Published:** 2023-10-05

**Authors:** Shuaijun Li, Zijun Xu, Zaipeng Yu, Yanrong Fu, Xiangping Su, Bingzhang Zou, Sirong Wang, Zhiqun Huang, Xiaohua Wan

**Affiliations:** 1 Key Laboratory for Humid Subtropical Eco-Geographical Processes of the Ministry of Education, Institute of Geographical Sciences, Fujian Normal University, Fuzhou, 350007 Fujian China; 2 Baisha National Forest Farm of Fujian Province, Shanghang, 364205 Fujian China

**Keywords:** Forest ecology, Forestry

## Abstract

In terrestrial ecosystems, leaf litter is the main source of nutrients returning to the soil. Understanding how litter decomposition responds to stand age is critical for improving predictions of the effects of forest age structure on nutrient availability and cycling in ecosystems. However, the changes in this critical process with stand age remain poorly understood due to the complexity and diversity of litter decomposition patterns and drivers among different stand ages. In this study, we examined the effects of stand age on litter decomposition with two well-replicated age sequences of naturally occurring secondary forests and Chinese fir (*Cunninghamia lanceolata*) plantations in southern China. Our results showed that the litter decomposition rates in the secondary forests were significantly higher than those in the Chinese fir plantations of the same age, except for 40-year-old forests. The litter decomposition rate of the Chinese fir initially increased and then decreased with stand age, while that of secondary forests gradually decreased. The results of a structural equation model indicated that stand age, litter quality and microbial community were the primary factors driving nutrient litter loss. Overall, these findings are helpful for understanding the effects of stand age on the litter decomposition process and nutrient cycling in plantation and secondary forest ecosystems.

## Introduction

In terrestrial ecosystems, leaf litter is the main source of nutrients returning to the soil^1–3^. Litter decomposition plays a crucial role in regulating nutrient cycling and organic matter turnover within forest ecosystems^4,5^. At a local scale, litter quality and decomposers are the key factors influencing litter decomposition and nutrient release^6–8^. As forest stand age progresses, the microclimate, soil properties, vegetation composition and microbial community undergo changes, exerting a significant influence on litter decomposition and nutrient release^9–11^. Previous studies have shown that the decomposition rate of litter will not change monotonically due to the ratio of litter carbon to nitrogen first increasing and then decreasing with stand age^12,13^. However, other studies have indicated that the litter decomposition rate decreased gradually as the initial carbon content of litter increased with stand age^14^. These inconsistent results highlight the complexity of changing patterns and driving factors of litter decomposition across different stand ages^14^. Therefore, exploring the dynamics and mechanisms of litter decomposition at various stand ages is crucial for gaining a comprehensive understanding of nutrient cycling in forest ecosystems.

Litter microorganisms and their activities are critical to litter decomposition^9,15,16^. The microbial community and enzyme activity within litter are highly sensitive to changes in nutrient availability and vegetation diversity that occur as forests age^10,17^. Evidence shows that both the microbial community and enzyme activity within litter tend to increase with stand age^17,18^. Moreover, studies have demonstrated that the microbial communities and enzyme activity within litter exhibit responses to variations in plant diversity and composition that arise from forest age^10,19^. In addition, the abundance and diversity of microorganisms tend to increase as forests age due to the relative humidity increasing during forest succession^20^.

In southern China, naturally occurring secondary forests and Chinese fir plantations are the dominant forest types, with cover areas reaching over 138 million ha and 11 million ha, respectively^21,22^. For our study, we established long-term age sequences in both Chinese fir plantations and naturally occurring secondary forests, with three replicates for each forest age. A two-year field litter decomposition experiment was conducted to evaluate the effects of litter quality and microbial changes with stand age on litter decomposition and nutrient release in the different forest types. We hypothesized that (1) compared to Chinese fir plantations, naturally occurring secondary forests would exhibit a higher litter decomposition rate and nutrient release than of the same age stage, and this scenario would be attributed to the higher litter nitrogen content in natural secondary forests. (2) The decomposition rate of litter would reach its peak at the middle stand age. Relative humidity and nutrient availability increase with stand age and provide favourable conditions for microbial richness and diversity, ultimately promoting litter decomposition^10,20,23^. (3) The nutrient release rate of a middle-aged forest is faster than that of a young forest and a mature forest, especially in the early stage of litter decomposition. Middle-aged forests with higher litterfall production and lower carbon–nitrogen ratio will accelerate the release of litter nutrients^24–26^.

## Results

### Dynamics of litter mass, C and N loss

**Figure 1:**
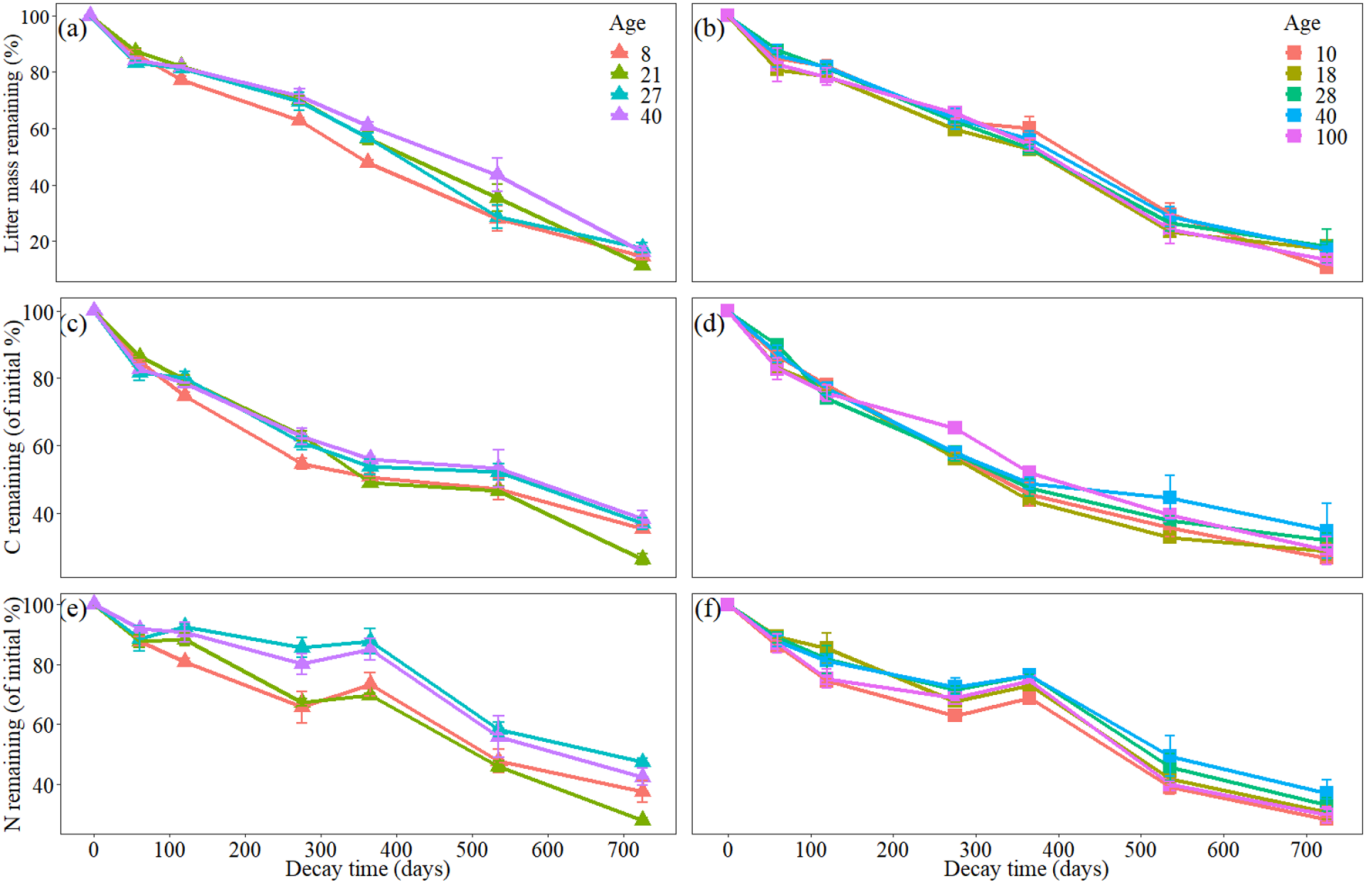
Litter mass, C and N remaining in litter from different stand age classes of Chinese fir (**a**,**c**,**e**) and naturally occurring secondary forest (**b**,**d**,**f**). Values represent the mean ± se.

At the early stage of the decomposition experiment, both Chinese fir and naturally occurring secondary forest exhibited rapid litter mass loss, with litter remaining at approximately 83.27–87.35% for Chinese fir and 80.5–87.43% for naturally occurring secondary forest at 60 days (Fig. 1a,b). Over the subsequent two months, the litter mass loss in both Chinese fir and naturally occurring secondary forest slowed. In the other decomposition stages, the rate of litter mass remaining continued to decline with fluctuations (Fig. 1a,b). At 275 and 365 days, the litter mass remaining in CF8 was significantly lower than that in the other Chinese fir stands (*P* < 0.05). At 535 days, both CF8 and CF27 had significantly lower litter mass remaining compared to that in CF40 (*P* < 0.05). At 275 days, the litter mass remaining in the primary forests (NF100) was significantly higher than that in NF10 (*P* < 0.05). At 365 days, the litter mass remaining in NF18 and NF28 was significantly lower than that in NF10 (*P* < 0.05).

The temporal pattern of litter carbon (C) and nitrogen (N) loss depended on forest type and stand age (Table 1). Specifically, in the Chinese fir stands, compared to CF27 and CF40, CF21 exhibited significantly faster leaf litter C and N loss (Fig. 1c,e). The leaf litter C and N losses of NF10 were significantly higher than those of NF18, NF28 and NF38 (Fig. 1d,f). In the middle stage of decomposition, there was an increase in the proportion of remaining N to initial nitrogen, suggesting slight net N immobilization in both Chinese fir and naturally occurring secondary forest (Fig. 1e,f).

**Table 1:** Results of a three-way repeated-measures ANOVA (*F* and *P* values) testing for the effects of forest type, stand age, decomposition time and their interactions on mass loss and leaf litter C and N loss from litterbags decomposed in soils in the Chinese fir and naturally occurring secondary forest stands.

Variation	df	Mass loss (%)^a^		k^b^		Litter C loss^a^		Litter N loss^b^	
		F	*P*	F	*P*	F	*P*	F	*P*
Forest type (F)	1	130.87	< 0.001	75.523	< 0.001	104.318	< 0.001	166.884	< 0.001
Stand age (A)	7	5.58	< 0.001	4.528	< 0.001	3.684	0.001	19.292	< 0.001
Time (T)	6	1865.39	< 0.001	255.057	< 0.001	1059.478	< 0.001	889.353	< 0.001
F × A	8	0.333	0.952	1.361	0.217	0.441	0.895	1.204	0.299
F × T	6	16.58	< 0.001	4.767	< 0.001	14.963	< 0.001	10.215	< 0.001
A × T	42	1.72	0.011	2.085	0.001	1.645	0.018	1.96	0.003
F × A × T	62	186	< 0.001	28.17	< 0.001	107.2	< 0.001	101.48	< 0.001

^a^Plot was not a significant random factor and was dropped from the model.

^b^Plot was a significant random factor and included in the model.

### Litter decomposition rate

The litter decomposition rates of the Chinese fir forest first increased and then decreased with stand age, whereas those of the naturally occurring secondary forest first decreased and then increased with stand age (Table 2). The decomposition rates of the naturally occurring secondary forest were significantly higher than those of Chinese fir forest, excluding the 40-year-old forest (*P* < 0.05). Among the Chinese fir stands, the litter decomposition rates in CF21 were significantly higher than those in CF8, CF27 and CF40 (*P* < 0.05). In the naturally occurring secondary forest, the decomposition rates in NF10, NF18 and NF100 were significantly higher than those in NF38 (*P* < 0.05).

**Table 2:** Models (*y* = *ae*^−*kt*^) for the relationship between mass remaining (*y*, %) of Chinese fir and naturally occurring secondary forest leaf litter and time (*t*, a).

	Regression equation	Decomposition constant (g g^−1^ a^−1^)	R^2^	T_50_(a)
CF8	*y* = 97.72*e*^−0.581*t*^	0.581Bb	0.991	1.15
CF21	*y* = 105.15*e*^−0.683*t*^	0.683Ba	0.969	1.09
CF27	*y* = 98.1*e*^−0.539*t*^	0.539Bc	0.981	1.26
CF40	*y* = 99.26*e*^−0.538*t*^	0.538Abc	0.977	1.27
NF10	*y* = 105.46*e*^−0.872*t*^	0.872Aa	0.982	0.87
NF18	*y* = 101.45*e*^−0.832*t*^	0.832Aab	0.973	0.85
NF28	*y* = 103.51*e*^−0.762*t*^	0.762Abc	0.992	0.96
NF38	*y* = 98.58*e*^−0.609*t*^	0.609Ac	0.995	1.12
NF100	*y* = 104.56*e*^−0.823*t*^	0.823ab	0.965	0.9

T50(a) represents the time required for a 50% mass loss for the litter from different forest age sequences. The uppercase letters indicate significant differences under two forest types of similar forest age, while the lowercase letters indicate significant differences under different stand ages of the same forest type (*P* < 0.05).

### Changes in litter microbial community composition

**Figure 2:**
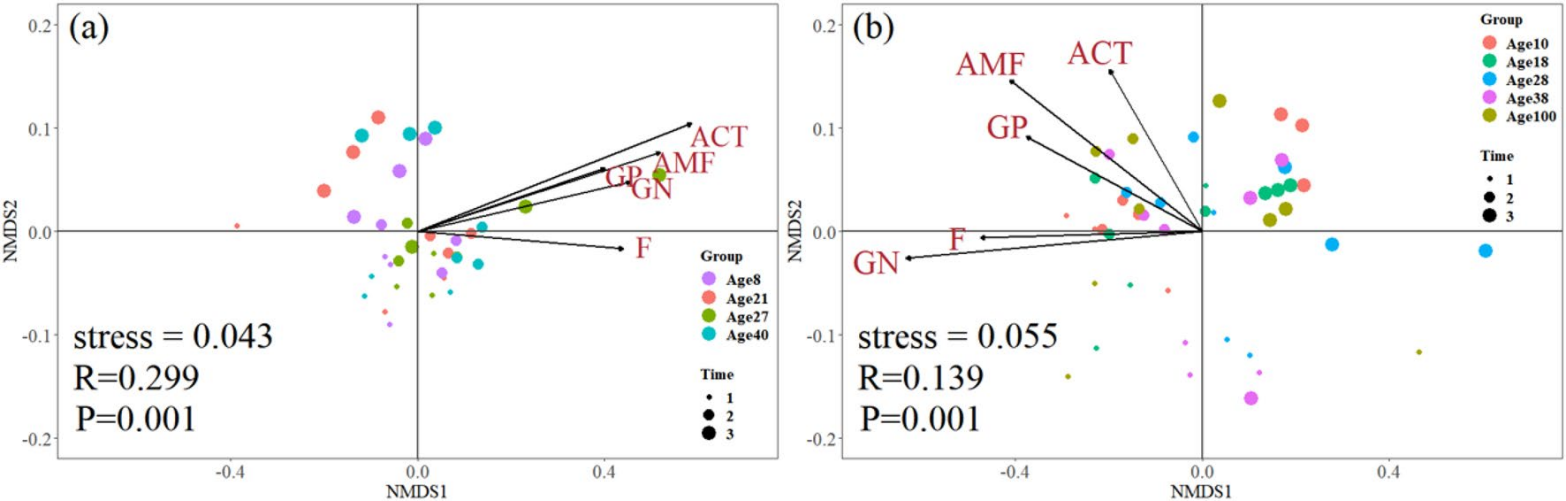
Nonmetric multidimensional scaling (NMDS) ordination of microbial community composition for Chinese fir (**a**) and naturally occurring secondary forest (**b**) during the decomposition period. The groups represent the stand age of Chinese fir and natural secondary forest; the time represents sampling dates (on the 0, 365th and 725th days). *Stress* Stress function in groups, *R* Similarities of microbial community, *P* Significance of similarity analysis of microbial community at different times, *GP* Gram-positive bacteria, *GN* Gram-negative bacteria, *F* Fungi, *AMF* Arbuscular mycorrhizal fungi, *ACT* Actinomycetes.

The composition of the microbial community within the litter exhibited significant differences throughout the decomposition process, showing dispersion effects of different forest types and stand ages at the beginning of decomposition, and they gradually converged at the later stage of litter decomposition (Fig. 2). Both Chinese fir and naturally occurring secondary forest displayed a dominance of fungi at the early stages, while the proportions of gram-positive bacteria, gram-negative bacteria, arbuscular mycorrhizal fungi, fungi and actinomycetes increased at the middle stage (Fig. 2). However, the composition of the microbial community converged with the decomposition process for leaf litter from the Chinese fir and naturally occurring secondary forest stands (Fig. 2).

### Factors that control the litter decomposition rate, mass and nutrient loss

The results of the linear mixed-effect model indicated that litter decomposition was synergistically controlled by forest age, soil environment, litter quality, and microorganisms. The results of the optimal linear mixed effect model for the litter decomposition rate indicated that the litter decomposition rate was mainly regulated by the contents of soil N, litter N, lignin, cellulose and β-N-acetyl-glucosaminidase (NAG) and the F: (GP + GN) ratio (*P* < 0.05, Supplementary Table 1). The combination of the above factors with cellobiohydrolase (CBH), arbuscular mycorrhizal fungi (AMF) and soil pH explained 37% of the variation in the litter decomposition rate. Moreover, the results of the optimal linear mixed effect model demonstrated that litter mass and nutrient loss are mainly regulated by the soil environment, litter quality, microbial community and their interaction. The ratio of soil C:N and total PLFAs showed a significantly positive effect on the remaining leaf litter mass, while litter N, lignin:litter N, AMF, fungi and GP had a significantly negative effect (*P* < 0.05, Supplementary Table 2). The litter C, NAG and F:(GP + GN) ratio showed a significantly negative effect on litter C loss, whereas the litter N, cellulose, lignin, AMF, fungi and SM showed a significantly positive effect (*P* < 0.05, Supplementary Table 3). The litter N, lignin, cellulose, AMF, fungi and GP:GN ratio showed a significantly positive effect on litter N loss, while the litter C, NAG, ammoniacal nitrogen (${\text{NH}}_{{4}}^{ + }$) and F:(GP + GN) ratio had a significantly negative effect (*P* < 0.05, Supplementary Table 4).

**Figure 3:**
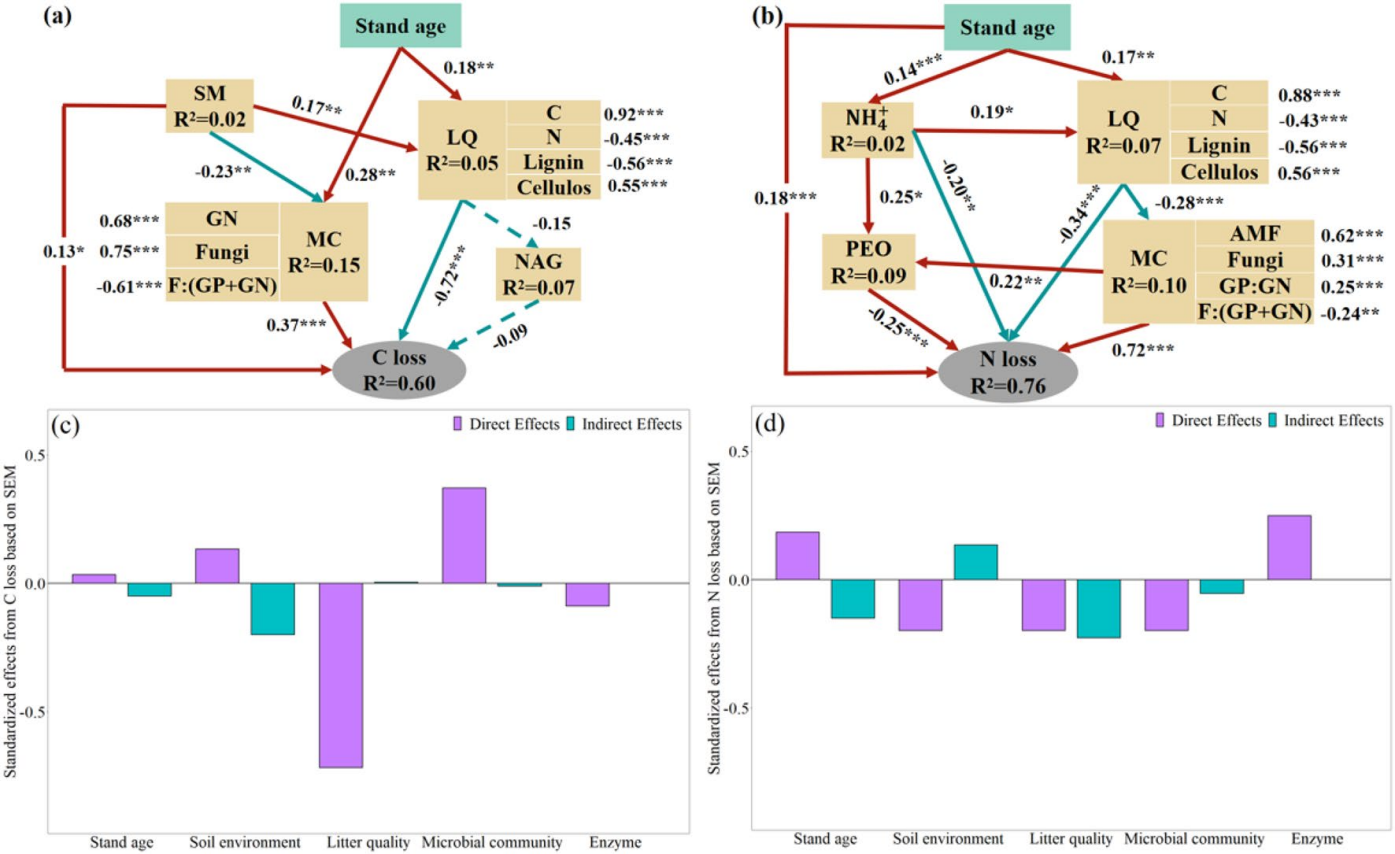
Structural equation model showing the effects of stand age, soil environment, soil nutrients, litter quality, microbial community and enzyme activity on litter C and N loss (N = 189). (**a**,**b**) Path diagrams of factors influencing litter C and N stocks. (**a**,**b**) Summed direct and indirect effects. Solid red and green arrows represent positive and negative relationships, respectively. Dashed arrows represent no significant effects. Numbers adjacent to arrows are standardized path coefficients, analogous to relative regression weights. (***P < 0.001; **P < 0.01; *P < 0.05). R^2^ represents the proportion of variance explained for every response variable in the model. The goodness-of-fit statistics for panels (**a**,**b**) are *P* = 0.306, GFI = 0.98, RMSEA = 0.027 and *P* = 0.421, GFI = 0.974, RMSEA = 0.013, respectively. *SE* Soil environment, *LQ* Litter quality, *MC* Microbial community, *EN* Enzyme, *SM* Soil moisture, *C* Litter C, *N* Litter N, *GN* Gram-negative bacteria, *F:(GP* + *GN)* Fungal-bacterial ratio, *AMF* Arbuscular mycorrhizal fungi, *NAG* β-N-acetyl-glucosaminidase, *PEO* Peroxidase.

The results of structural equation modelling indicated that the soil environment, litter quality, microbial community, enzyme activity and their interactions could directly or indirectly drive litter nutrient loss (Fig. 3a,b). The litter quality and microbial community were the main factors that directly controlled litter C and N loss (*P* < 0.001, Fig. 3a,b). Litter quality showed a significantly negative direct effect on litter C and N loss, whereas the microbial community had a significant direct positive effect (Fig. 3a,b). At the same time, stand age and soil environment indirectly affected litter C and N loss by affecting litter quality (Fig. 3). The effect of litter enzyme activity on litter C and N loss was not significant.

## Discussion

Our results showed that the litter decomposition rate of the secondary forest was significantly higher than that of the Chinese fir plantation at the same age, except for the 40-year-old forest, which was consistent with our first hypothesis. Previous studies have indicated that the decomposition rate of broad-leaved species is significantly higher than that of conifer species^27–29^. Our results support this conclusion due to the lower litter N content and the presence of more recalcitrant substances (i.e., lignin and cellulose) in the Chinese fir plantations (Table S5). Furthermore, as stand age increases, changes in plant species and composition have a positive effect on decomposition rates^30^. Previous studies at our site have suggested that understorey vegetation diversity is higher in naturally occurring secondary forests and primary forests than in Chinese fir plantations^31^. In addition, during the process of litter decomposition, the mixture of litter with different qualities will interact and produce positive nonadditive mixture effects, thus promoting litter decomposition^32–34^. These factors collectively contribute to the accelerated litter decomposition rate in naturally occurring secondary forests and primary forests.

The quality of litter exerts an important influence on the litter decomposition rate^35,36^. Consistent with our second hypothesis, the decomposition rate of the litter in the middle-aged Chinese fir forests was higher than that in the younger and older forests. The characteristics of litter with a higher N content and lower C:N ratio in the middle-aged forests were the main reasons for the high decomposition rate^35,37^. Additionally, during the process of forest development, the forest self-thinning effect enlarges the forest gap and enhances the photodegradation of litter, thereby accelerating the decomposition of litter^5,38,39^. Moreover, the relative humidity tends to increase in middle-aged forests with a higher diversity of understorey vegetation, which further accelerates the decomposition of litter^20,31^.

For the age sequence of the secondary forest, the decomposition rate of litter decreased with stand age and increased in the primary forest, which was contrary to our second hypothesis. The increasing canopy cover in a secondary forest as the forest develops and the subsequent decrease in understorey biomass can slow litter decomposition^33,40,41^. Previous studies conducted on the age sequences of secondary forests in tropical regions have also indicated an increase in relative humidity with stand age, accompanied by a decrease in light availability and soil temperature^20^. The variation in environmental factors such as forest canopy cover, vegetation type, soil temperature and soil moisture with forest stand age can reduce nonadditive mixture effects, which can hinder litter decomposition^20,42^. Variations in stand vegetation type, age scale and study location may account for the differences described above^11,13,42^. However, previous studies have shown that forest type exerts an important influence on litter decomposition^42^. Our findings support this notion by revealing distinct patterns of litter decomposition rates with the age sequences of secondary forests and Chinese fir forests, further highlighting the importance of forest type in litter decomposition.

In both the Chinese fir and secondary forest age sequences, the nutrient release rate of middle-aged forest was faster than young and mature forests, which was consistent with our third hypothesis. In our study, middle-aged forests with higher litterfall production and lower carbon–nitrogen ratio accelerate the release of litter nutrients^24,35^. Litter C and N are necessary nutrients for soil microbial activities are easily absorbed and utilized by microorganisms, and quickly released in the early stages of decomposition^25^. It has been well documented by several researchers that the general trend of litter decomposition is initially rapid and then slow^33,43,44^. The present study found that the rate of nitrogen release was faster in the younger forest, possibly due to the high demand for nitrogen by vegetation in the early stages of forest regeneration to support their growth^26^. Previous studies have also suggested that temporal patterns of litter C and N loss are influenced by stand age^11,30^, and the results of our research also support this conclusion. The results of the structural equation model showed that stand age had an indirect effect on litter C and N loss by increasing litter nitrogen and/or microbial biomass. Furthermore, lignin had a significant negative effect on the release of carbon and nitrogen from litter, which was consistent with previous studies^36^.

Changes in forest stand age may be paralleled by shifts in microbial community composition^10,45^. Our results demonstrated that there were differences in the microbial community composition at the initial stage of litter decomposition among the different stand ages of the naturally occurring secondary forests and Chinese fir, although these differences were not significant (Fig. 3). The ability to metabolize easily accessed carbon is believed to be universal to various microorganisms^46^. In the early stage of decomposition, easily accessed or unbound carbon accounts for a high proportion of the initial composition and cannot be a limiting factor for microbial activities^47,48^. At the later stage of decomposition, microbial community composition in litter converged in the two forest types with different stand ages. This finding may be attributed to the depletion of labile substrates during further decomposition or their binding with other compounds, making them less available for microbial utilization^48^.

Enzymatic activities are generated in response to limited substrate and nutrient availability, facilitating nutrient acquisition during decomposition^9^. Our results showed that the soil environment did not have a direct effect on enzyme activities. Previous studies have indicated that enzyme activity can occur over a broad temperature range or based on the production of multiple isoenzymes, each with different temperature optima but with similar affinity to the substrate^49^. Other studies have shown that the decoupling between enzyme activities and the soil environment may indicate that microbial growth is not primarily limited by nutrient availability^50^. In addition, the accumulation of microbial biomass always delays the increase in enzyme activity^9^, which further supports our research results.

## Conclusions

In conclusion, the effects of forest stand age on litter decomposition and nutrient release dynamics in naturally occurring secondary forests and Chinese fir plantations were tested in this study. Our results suggest that forest age and forest type have a significant influence on litter quality, thus affecting nutrient cycling. The nutrient cycle of Chinese fir plantations first increased and then decreased with stand age and slowed with secondary forest succession. Furthermore, our results also showed that the nutrient turnover in the secondary forest was significantly faster than that in the Chinese fir plantations of the same age, except for the 40-year-old forest. These findings are helpful for understanding the effects of stand age on the process of litter decomposition and nutrient cycling in plantation and secondary forest ecosystems. Unfortunately, our research lacks the inclusion of older forests, which may have an impact on the research results. Therefore, it is necessary to carry out further research in forest age series containing more forest age stages.

## Methods

### Study area and experimental design

This experiment was conducted in naturally occurring secondary forests and Chinese fir (*C. lanceolata*) plantations, which are located in Baisha Forest Farm (116° 20′ 00″–116° 40′ 57″ E; 24° 52′ 01″–25° 16′ 59″ N) in Fujian Province, Southeast China. According to the Köppen climate classification, the climate is monsoon-influenced humid subtropical, with long hot and humid summers and short mild winters. The mean annual precipitation is 1646 mm, and the mean annual temperature is 20.1 °C (lowest in January, i.e., 6.7 °C; highest in July, i.e., 29.1 °C), with a frost-free period of 277 days^31^. The elevation ranges from 453 to 875 m. According to the IUSS Working Group WRB 2014, the soil type in the study area is Oxisol^51^, and the soils were developed from granite. Natural secondary forest species are mainly dominated by *Castanopsis carlesii*, *Castanopsis fargesii*, *Schima Superba*, *Castanopsis faberi*, *Liquidambar formosana*, *Sloanea sinensis*, etc.

In May 2019, we selected four stages of Chinese fir plantations and natural secondary forests, with 8-, 21-, 27-, and 40-year-old Chinese fir stands (hereafter called CF8, CF21, CF27, and CF40, respectively) and approximately 10-, 18-, 28-, and 38-year-old natural secondary forests (hereafter called NF10, NF18, NF28, and NF38, respectively). In addition, we selected primary forests (> 100 yr) that were undisturbed by humans for more than centuries (hereafter called NF100). The natural secondary forests were transformed from clear-cutting Chinese fir and Pinus massoniana plantations. There is no human intervention in the process of growth. We mainly determine the age of the secondary forest according to the logging records of the forest farm. Three replicated plots (20 × 30 m = 600 m^2^) were chosen for each stand age class, and replicated plots of the same stand age were at least 500 m apart. In addition, to prevent the edge effect, all experimental plots were at least 100 m away from the edge of farmland, road or other types of woodland. After the establishment of the sample plot, we investigated the stand characteristics and vegetation community composition of Chinese fir plantations and natural secondary forests with different stand ages (Table 3).

**Table 3:** The stand characteristics and vegetation community composition of Chinese fir plantations and natural secondary forests with different stand ages.

Plot	N	Stand density (stems/ha)	Mean DBH (cm)	Basal area (m^2^/ha)	Stand composition (% of stand basal area)						
					Chinese fir	*Castanopsis carlesii*	*Castanopsis fargesii*	*Schima superba*	*Castanopsis faberi*	*Castanopsis fissa*	Other^a^
CF8	3	3088 ± 804	12.28 ± 0.68	32 ± 2.95	97 ± 2	0	0	0	0	0	3 ± 2
CF21	3	1400 ± 352	19.81 ± 1.01	40.2 ± 7	93 ± 3.5	0	0	0	0	0	6 ± 3.5
CF27	3	1863 ± 444	23.22 ± 1.37	43.8 ± 8.45	96 ± 2	0	0	0	0	0	3 ± 2
CF40	3	1142 ± 291	27.85 ± 1.94	43.4 ± 5.3	92 ± 3	0	5 ± 1	0	0	0	3 ± 1.5
NF10	3	2739 ± 110	9.32 ± 2	23.2 ± 4.1	0	1 ± 0.5	14 ± 6	3 ± 1	0	20 ± 9.5	62 ± 14.5
NF18	3	2244 ± 283	10.9 ± 1.4	25.7 ± 2.4	3 ± 1	9 ± 8	46 ± 3.5	6 ± 4	4 ± 3.5	0	32 ± 4.5
NF28	3	2658 ± 240	11.1 ± 0.2	31.1 ± 3.7	2 ± 1	0	41 ± 5.5	10 ± 3.5	4 ± 4	4 ± 3.5	39 ± 5.5
NF38	3	1739 ± 255	12.1 ± 0.6	27.7 ± 2.1	3 ± 1	11 ± 9.5	34 ± 12.5	26 ± 20.5	4 ± 4	0	22 ± 6.5
NF100	3	1179 ± 109	16.4 ± 1.4	56.8 ± 6.8	0	58 ± 21	0	1 ± 0.5	8 ± 4.5	0	33 ± 17.5

The others’ category included *Cinnamomum micranthum, Pinus massoniana, Sloanea sinensis, Cyclobalanopsis glauca, Cinnamomum camphora, Lithocarpus glaber, Meliosma rigida, Machilus chekiangensis, Liquidambar formosana, Cinnamomum porrectum, Micromelum falcatum, Illicium lanceolatum, Rapanea neriifolia, Engelhardtia fenzlii, Machilus versicolora, Eurya loquaiana, Rhododendron henryi, Phyllostachys heterocycle, Phoebe bournei, Ormosia xylocarpa, Castanopsis tibetana, Castanopsis eyrei, Neolitsea aurata, Adinandra millettii, Illicium ternstroemioides* and *Machilus velutina.*

In July 2019, we collected fresh fallen litters by the litter traps and recording the plot number. Six litter traps were setted in each 20 × 30 m plot. It should be noted that the litter samples collected in this study do not include endangered species, so there are no ethical issues and other conflicts of interest. Litter samples collected from each plot were combined to represent one sample. Then, all leaf litters of each subplot were air-dried and put into litterbags (20 cm × 25 cm) made from polyvinyl screen with a mesh width = 1.5 mm. To alleviate the effect of the mesh size of the litter bag on the soil biota and insects, 16 small holes of 0.25 cm^2^ were evenly distributed on both sides of the litter bag to enable access for some soil biota and insects. Each litterbag was filled with 10 g of leaf litter from the Chinese fir plantations or naturally occurring secondary forests and placed on the surface of the plot corresponding to the recorded data. In each plot, 18 litterbags containing the leaf litter were tied to nylon lines to prevent losses.

### Sampling and analysis

The decomposition of leaf litter started in August 2019, and two bags were randomly selected from each subplot at 0, 60, 120, 275, 365, 535, and 725 days after the start of the litter decomposition experiment. At the same time, the soil temperature (ST) and moisture (SM) were measured by a time domain reflectometer (Model TDR350, Spectrum Company, USA). In total, 378 litterbags (9 stand ages × 3 replicates × 2 litterbags × 7 sampling events) were collected and brought back to the laboratory to prepare for subsequent experiments. We carefully removed soil particles and other nontarget plant material adhering to the remaining litter. Then, we gently rinsed the remaining litter using forceps and oven-dried it at 65 °C for at least 72 h to constant weight. Subsequently, we weighed the remaining leaf litter and calculated the mass loss of litter in each sample plot. The contents of litter C and N were determined by an elemental analyser (Elementar Vario EL III, Elementar, Germany). Extraction of lignin and cellulose from the litter was conducted by the acid detergent method.

In July 2019, 0–10 cm soil samples were collected and analysed for physical and chemical properties. In each plot, 10 soil cores were randomly sampled by using a soil auger with a 5 cm diameter. All soil samples were stored in coolers and brought back to the laboratory immediately. After removing rocks and visible plant debris, the soil samples were sieved through a 2 mm mesh and mixed. All samples were divided into two subsamples, with one stored at 4 °C for dissolved organic N (DON) and mineral N determination and the other subsample air dried for analyses of soil pH, total carbon (TC) and total nitrogen (TN). Soil mineral N and DON were measured by a method in previous research^52^. Soil pH was determined by a glass electrode pH meter (STARTER 300, OHAUS, US) with a water to soil ratio of 2.5:1^52^. Soil TC and TN were assessed on finely ground soil (< 0.20 mm) using an element analyser with a combustion furnace (Elementar Vario EL III, Elementar, Germany).

### Litter microbial community and enzyme activity

The litter microbial community was measured by phospholipid fatty acid (PLFA) analysis and extracted only from litterbags collected at 0, 365 and 725 days after the start of the experiment because of resource constraints. We extracted phospholipids from freeze-dried litter samples according to the methods in previous studies^48,53^. Fatty acids were extracted from 1 g of litter samples in a one-phase solvent consisting of a 2:1:0.8 mixture of methanol, chloroform and phosphate. The phospholipid fraction was collected and transformed to fatty acid methyl esters (FAMEs) by methylation. The FAMEs were identified by gas chromatography with the MIDI Sherlock Microbial Identification System (MIDI, Inc.) and by coelution with standards. The concentrations of the PLFAs were calculated according to the internal standard concentration of 19:0. Individual PLFAs were identified using standard nomenclature. Gram-positive bacteria (GP) were assessed by i14:0, i15:0, a15:0, i16:0, i17:0 and a17:0, whereas gram-negative bacteria (GN) were represented by 16:1 w9c, 16:1 w7c, cy17:0, 18:1 w7c and 18:1 w5c^54,55^. Actinomycetes (ACT) were evaluated by 10Me16:0, 10Me17:0 and 10Me18:0^56^. The fungi (F) were represented by 18:1ω9c and 18:2ω6c^57,58^. The arbuscular mycorrhizal fungi (AMF) were assessed by 16:1ω5c^57^.

Fresh litter samples for each stand age and harvesting time were used to analyse the activities of four hydrolytic enzymes, cellobiohydrolase (CBH), beta-glucosidase (BG), β-N-acetyl-glucosaminidase (NAG), and acid phosphatase (AP), and two oxidase enzymes, phenol oxidase (PHO) and peroxidase (PEO), involved in the microbial acquisition of C, N, P and refractory substances^15,59^. Freshly cut litter samples (1.5 g) were added to a 125 ml conical flask with 50 mmol/L acetate buffer (pH = 5) and stirred with a magnetic stirrer. The mixture was stirred for 10 min, allowed to stand, and then transferred to a clean beaker for further use. Aliquots of 200 µl of suspension were dispensed into 96-well microplates, and then, 50 µl of substrate solution was added at a concentration of 200 µmol substrate g^−1^ sample. Finally, the microplates were incubated at 25 °C with hydrolytic enzyme for 4 h and oxidase for 20 h. After incubation, the reaction of the hydrolytic enzyme was terminated by adding a 10 µl aliquot of NaOH to each well^9^. The enzyme activities were measured by a multifunction microplate reader (Spectra Max M5, Molecular Devices, USA).

### Litter decomposition rate

Litter mass remaining refers to the loss of the dry weight of litter after a period of decomposition, denoted as R (%), and the equation was as follows^60^:

$${\text{R}}\left( \% \right) = {\text{M}}_{{\text{t}}} /{\text{M}}_{0} \times 100\%$$

where M_0_ (10 g) is the initial dry weight of litter in each bag and M_t_ (g) is the dry weight of litter at time t. The litter decomposition rates (k) were determined by an exponential decay model^61^:

$${\text{M}}_{{\text{t}}} = {\text{M}}_{0} \times {\text{e}}^{{ - {\text{kt}}}}$$

where M_0_ and M_t_ are the same as above. The content of organic C and N in litter was determined by potassium dichromate and sulfuric acid heating^62^, the Kjeldahl method^63^, and molybdenum antimony colorimetry^64^.

### Statistical analysis

First, a linear mixed effects model (LMM) was performed to evaluate the effect of forest type, stand age, time and their interactions on leaf litter mass remaining, C and N loss with forest type, stand age and sample time as fixed factors and plot as a random-effect factor using the package *lme4* in R v.4.2.0 (R Development Core Team, 2022). Nonmetric multidimensional scaling (NMDS) based on Bray‒Curtis distance was performed to assess the multivariate analysis of the microbial community composition of litter for the Chinese fir and naturally occurring secondary forest stands.

Second, another linear mixed effects model (LMM) was carried out to identify the main drivers of the litter decomposition rate and nutrient loss of forest ecosystems by examining biophysical parameters previously shown to influence decomposition processes. The initial model included stand age, soil environment, litter quality, microbial community and enzyme activity parameters with plot as a random effect factor. To prevent overfitting, we deleted the variable with a variance expansion factor (VIF) > 5 on the premise that the stand age was selected into the model. Afterwards, the optimal model was selected by using the ‘*dredging*’ function of the *MuMIn* package according to the Akaike information criterion (AIC) fitted with maximum likelihood.

Finally, structural equation modelling (SEM) was performed to test the effect of stand age, soil environment, litter quality, microbial community and enzyme activities on litter C and N loss by using the package *lavaan* in R. Soil environment (including pH, DON, TC, TN, etc.), litter quality (including C, N, lignin, cellulose, etc.), microbial community (including GN, fungi, AMF, etc.) and enzyme activity (including CBH, BG, NAG, AP, etc.) were included in the model as composite variables. We proposed a priori SEM model to evaluate the direct and indirect effects of stand age and the above-mentioned composite variables on litter C and N loss. We retained the soil environment (SM or ${\text{NH}}_{{4}}^{ + }$) and enzyme activity (NAG or PEO) indicators in the final SEM models, which had significant effect on litter quality, microbial community, and enzyme activities. We conducted hypothetical causal models that included all possible paths and improved the model fitting by modifying parameters (Supplementary Fig. 1). The final model was selected according to goodness-of-fit statistics and the Akaike information criterion (AIC) among several models^65^. We used the chi-square test (*P* > 0.05), GFI (GFI > 0.95) and RMSEA (RMSEA < 0.05) to indicate a good fit of the structural equation models. All statistical analyses and graphs in this article were conducted using R software v.4.2.0 (R Development Core Team, 2022).

### Supplementary Information

Supplementary Information.

## Data Availability

The datasets analysed during the current study are listed in Supplementary Materials. All datasets generated during the current study are available immediately after its publication.
