# Supplementary material for: Litter decomposition and nutrient release are faster under secondary forests than under Chinese fir plantations with forest development
Source: Sci Rep. 2023 Oct 5;13:16805. doi: 10.1038/s41598-023-44042-5 (PMC10555996; doi:10.1038/s41598-023-44042-5)
Supplement: Supplementary file 1 — Supplementary Information. [file 41598_2023_44042_MOESM1_ESM.docx]

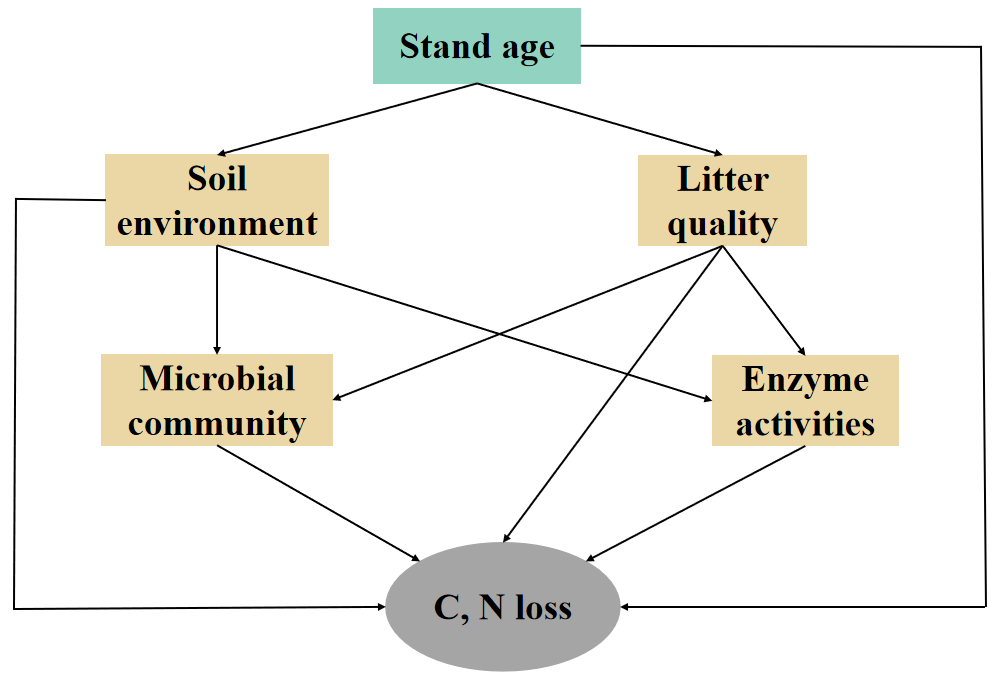


Figure S1. Suggested causal pathways of direct and indirect effects of stand age, soil environment, litter quality, microbial community and enzyme activities on litter decomposition and C or N loss.

Table S1. Results of an optimal model for litter decomposition rate. Estimates obtained from mixed effect model with plot as a random factor. R^2^ marginal=0.37; R^2^ conditional = 0.37.

|  | Estimate | Std. Error | t | *p* |
| --- | --- | --- | --- | --- |
| Intercept | 0.444 | 0.012 | 36.113 | < 0.001 *** |
| Litter N | 0.132 | 0.022 | 6.072 | < 0.001 *** |
| Lignin | 0.146 | 0.019 | 7.701 | < 0.001 *** |
| Cellulose | 0.05 | 0.016 | 3.099 | 0.002 ** |
| F:(GP + GN) | -0.033 | 0.013 | -2.533 | 0.012* |
| AMF | 0.003 | 0.013 | 0.23 | 0.818 |
| NAG | -0.043 | 0.02 | -2.171 | 0.031 * |
| CBH | -0.015 | 0.019 | -0.802 | 0.424 |
| DON | -0.038 | 0.018 | -2.125 | 0.035 * |
| TN | 0.034 | 0.016 | 2.124 | 0.035 * |
| pH | 0.029 | 0.015 | 1.868 | 0.063 |

Table S2. Results of an optimal model for litter mass remaining. Estimates obtained from mixed effect model with plot as a random factor. R^2^ marginal=0.66; R^2^ conditional = 0.70.

|  | Estimate | Std. Error | t | *p* |
| --- | --- | --- | --- | --- |
| Intercept | 1.742 | 0.029 | 60.731 | < 0.001 *** |
| Litter N | -0.184 | 0.08 | -2.305 | 0.023* |
| Lignin:Litter N | -0.219 | 0.05 | -4.366 | < 0.001 *** |
| AMF | -0.487 | 0.07 | -6.928 | < 0.001 *** |
| Fungi | -0.13 | 0.031 | -4.19 | < 0.001 *** |
| GN | -0.026 | 0.033 | -0.767 | 0.444 |
| TOTAL | 0.677 | 0.068 | 10.007 | < 0.001 *** |
| GP | -0.326 | 0.087 | -3.75 | < 0.001 *** |
| NAG | 0.045 | 0.026 | 1.722 | 0.087 |
| PEO | -0.045 | 0.024 | -1.866 | 0.064 |
| Soil C: N | 0.256 | 0.072 | 3.566 | < 0.001 *** |

Table S3. Results of an optimal model for litter C loss. Estimates obtained from the mixed effect model with plot as a random factor. R^2^ marginal=0.75; R^2^ conditional = 0.75.

|  | Estimate | Std. Error | t | *p* |
| --- | --- | --- | --- | --- |
| Intercept | 3.156 | 0.051 | 61.588 | < 0.001 *** |
| Litter C | -0.557 | 0.067 | -8.342 | < 0.001 *** |
| Litter N | 0.843 | 0.086 | 9.852 | < 0.001 *** |
| F:(GP + GN) | -0.155 | 0.063 | -2.461 | 0.015 * |
| Cellulose | 0.288 | 0.068 | 4.232 | < 0.001 *** |
| Lignin | 1.036 | 0.074 | 14.05 | < 0.001 *** |
| AMF | 0.196 | 0.063 | 3.126 | 0.002 ** |
| Fungi | 0.081 | 0.065 | 1.244 | 0.215 |
| NAG | -0.226 | 0.059 | -3.807 | < 0.001 *** |
| SM | 0.117 | 0.059 | 1.98 | 0.049 * |

Table S4. Results of an optimal model for litter N loss. Estimates obtained from mixed effect model with plot as a random factor. R^2^ marginal=0.68; R^2^ conditional = 0.68.

|  | Estimate | Std. Error | t | *p* |
| --- | --- | --- | --- | --- |
| Intercept | 2.88 | 0.056 | 51.626 | < 0.001 *** |
| Litter C | -0.456 | 0.072 | -6.374 | < 0.001 *** |
| Litter N | 0.668 | 0.09 | 7.421 | < 0.001 *** |
| Lignin | 0.866 | 0.087 | 9.997 | < 0.001 *** |
| Cellulose | 0.192 | 0.075 | 2.552 | 0.012 * |
| F:(GP + GN) | -0.193 | 0.072 | -2.671 | 0.008 ** |
| AMF | 0.217 | 0.064 | 3.38 | < 0.001 *** |
| Fungi | 0.153 | 0.068 | 2.236 | 0.027 * |
| GP: GN | 0.155 | 0.075 | 2.079 | 0.039 * |
| NAG | -0.207 | 0.066 | -3.147 | 0.002 ** |
| PEO | 0.098 | 0.06 | 1.646 | 0.102 |
| $\text{NH}_{\text{4}}^{\text{+}}$ | -0.15 | 0.061 | -2.442 | 0.016 * |

Table S5. Initial litter quality parameters of Chinese fir and naturally occurring secondary forest leaf litter. Different letters in the column indicate significant (p < 0.05) differences between the stand age classes. C: litter carbon; N: litter nitrogen. CF: Chinese fir plantation; NF: naturally occurring secondary forest. 8, 21, 27, 40, 10, 18, 28, 38, and 100 represent different forest stand ages.

| Stand age classes | C  (g kg^-1^) | N  (g kg^-1^) | C/N | Cellulose  (%) | Lignin  (%) |
| --- | --- | --- | --- | --- | --- |
| CF8 | 497.37±1.65a | 6.8±0.26ab | 73.32±2.86b | 19.83±2.64a | 32.27±3.39a |
| CF21 | 499.71±2.71a | 8.38±0.07a | 59.62±0.46c | 22.2±0.88a | 34.73±2.22a |
| CF27 | 508.93±7.31a | 6.94±0.61ab | 77.38±3.63ab | 20.29±0.55a | 35.9±0.28a |
| CF40 | 508.14±4.05a | 5.3±0.87b | 85.42±3.13a | 17.93±0.82a | 32.36±2.95a |
| NF10 | 488.21±0.88a | 11.29±0.53b | 43.43±2.04a | 16.71±1.62a | 20.79±0.84a |
| NF18 | 484.51±0.29a | 13.75±0.23ab | 35.26±0.62ab | 13.87±1.82a | 14.9±1.58a |
| NF28 | 489.13±2.11a | 11.35±0.75b | 43.48±2.89a | 17.9±1a | 19.37±1.35a |
| NF38 | 442.09±7.73b | 12.64±0.44b | 35.1±1.75ab | 15.42±1.01a | 20.58±0.97a |
| NF100 | 491.66±3.58a | 15.58±0.13a | 31.56±0.49b | 18.73±0.38a | 15.25±0.32a |

Table S6. Mean value of enzyme activities (CBH, BG, AP, NAG, PHO and PEO) in the Chinese fir plantations and naturally occurring secondary forests (values are averaged over seven sampling timepoints). Error bars are standard errors. CBH: cellobiohydrolase, BG: beta-glucosidase, AP: acid phosphatase, NAG: β-N-acetyl-glucosaminidase, PHO: phenol oxidase, PEO: peroxidase.

| Stand | CBH | BG | AP | NAG | PHO | PEO |
| --- | --- | --- | --- | --- | --- | --- |
| CF8 | 111±18 | 795±102 | 3969±301 | 378±33 | 176±57 | 513±45 |
| CF21 | 583±148 | 2153±374 | 3887±788 | 649±161 | 245±60 | 1625±541 |
| CF27 | 455±243 | 1703±618 | 1083±354 | 759±264 | 598±13 | 403±47 |
| CF40 | 238±30 | 1297±214 | 2615±280 | 537±126 | 36±1 | 689±18 |
| NF10 | 785±267 | 2504±492 | 3477±561 | 2022±271 | 290±81 | 304±141 |
| NF18 | 322±99 | 1637±305 | 3373±472 | 1562±501 | 74±15 | 164±37 |
| NF28 | 568±330 | 1635±694 | 3266±275 | 935±309 | 60±8 | 336±30 |
| NF38 | 154±64 | 1007±239 | 2985±164 | 895±383 | 69±13 | 316±57 |
| NF100 | 1589±220 | 3282±357 | 3315±498 | 2506±337 | 314±6 | 415±89 |
